# Dactinomycin induces complete remission associated with nucleolar stress response in relapsed/refractory *NPM1*-mutated AML

**DOI:** 10.1038/s41375-021-01192-7

**Published:** 2021-03-02

**Authors:** Ilaria Gionfriddo, Lorenzo Brunetti, Federica Mezzasoma, Francesca Milano, Valeria Cardinali, Roberta Ranieri, Alessandra Venanzi, Sara Pierangeli, Calogero Vetro, Giulio Spinozzi, Erica Dorillo, Hsin Chieh Wu, Caroline Berthier, Raffaella Ciurnelli, Melanie J. Griffin, Claire E. Jennings, Enrico Tiacci, Paolo Sportoletti, Franca Falzetti, Hugues de Thé, Gareth J. Veal, Maria Paola Martelli, Brunangelo Falini

**Affiliations:** 1 Hematology, Center for Research in Hemato-Oncology (CREO), University of Perugia, Perugia, Italy; 2 Hematology, A.O. “Policlinico S. Marco”, Catania, Italy; 3 INSERM U944 and 1050, IRSL, University of Paris and PSL, Hôpital St. Louis and Collége de France, Paris, France; 4 Newcastle University Centre for Cancer, Newcastle University, Newcastle, UK

**Keywords:** Translational research, Chemotherapy

## Abstract

Acute myeloid leukemia (AML) with mutated *NPM1* accounts for one-third of newly diagnosed AML. Despite recent advances, treatment of relapsed/refractory *NPM1*-mutated AML remains challenging, with the majority of patients eventually dying due to disease progression. Moreover, the prognosis is particularly poor in elderly and unfit patients, mainly because they cannot receive intensive treatment. Therefore, alternative treatment strategies are needed. Dactinomycin is a low-cost chemotherapeutic agent, which has been anecdotally reported to induce remission in *NPM1*-mutated patients, although its mechanism of action remains unclear. Here, we describe the results of a single-center phase 2 pilot study investigating the safety and efficacy of single-agent dactinomycin in relapsed/refractory *NPM1*-mutated adult AML patients, demonstrating that this drug can induce complete responses and is relatively well tolerated. We also provide evidence that the activity of dactinomycin associates with nucleolar stress both in vitro and in vivo in patients. Finally, we show that low-dose dactinomycin generates more efficient stress response in cells expressing NPM1 mutant compared to wild-type cells, suggesting that *NPM1*-mutated AML may be more sensitive to nucleolar stress. In conclusion, we establish that dactinomycin is a potential therapeutic alternative in relapsed/refractory *NPM1*-mutated AML that deserves further investigation in larger clinical studies.

## Introduction

The *NPM1* gene encodes for a multifunctional nucleolar chaperone [1, 2], that continuously shuttles between nucleus and cytoplasm [3, 4]. *NPM1* is mutated in approximately one-third of newly diagnosed acute myeloid leukemia (AML) cases [5]. *NPM1* mutations are usually heterozygous insertions in the last exon of the gene [5, 6] that cause the loss of the nucleolar localization signal and generation of a novel nuclear export signal [7, 8]. These changes result in aberrant delocalization of the NPM1 mutant in the nucleoplasm and in the cytoplasm that is, in turn, responsible for leukemogenesis through a yet unclear mechanism [9].

Because of its specific features [9], *NPM1*-mutated AML has been included as distinct entity in the World Health Organization (WHO) classification of lympho-hematopoietic tumors [10]. Criteria for the diagnosis, risk stratification and monitoring of measurable residual disease (MRD) of this AML entity are now well established [9–13]. Currently, its treatment still relies on standard chemotherapy (CHT) plus a FLT3 inhibitor (only in cases with mutated *FLT3*) with or without allogeneic hematopoietic stem cell transplantation (allo-HSCT), depending on the predicted relapse risk [11]. More recently, the combination of venetoclax plus hypomethylating agents or low-dose cytarabine [11, 14–16] has demonstrated promising clinical activity, particularly in patients unfit for intensive CHT. However, the majority of older patients with *NPM1*-mutated AML eventually relapse and die of progressive disease, clearly indicating that new therapeutic strategies are needed for this AML entity.

Actinomycin D (dactinomycin) is known to inhibit RNA Polymerase I (RNA Pol I) [17–19], depleting nucleoli of ribosomal RNA (rRNA) and inducing nucleolar stress response [20]. NPM1 is regarded as a nucleolar stress sensor [9, 21]. Specifically, following RNA Pol I inhibition, NPM1 is released [22] together with other nucleolar proteins (e.g., ribosomal proteins) [23] from the nucleolus to the nucleoplasm [20]. These molecules, including NPM1, bind to and inhibit the ubiquitin-ligase HDM2 leading to the consequent increase of TP53 levels.

We hypothesized that higher NPM1 levels in the nucleoplasm of *NPM1*-mutated AML cells (secondary to the aberrant localization of mutant NPM1 as well as, through heterodimerization, of wild-type NPM1) may facilitate nucleolar stress response and increase sensitivity to dactinomycin in *NPM1*-mutated AML cells [24]. Moreover, TP53 genetic abnormalities are extremely rare in *NPM1*-mutated AML [25, 26], implying that signals downstream of nucleolar stress response are preserved in leukemic cells, further supporting the use of dactinomycin in this setting.

Dactinomycin is currently used, alone or in combination with other drugs, to treat different types of solid tumors, both in adult and pediatric patients [27]. Main side effects of dactinomycin include dose-dependent myelosuppression, gastrointestinal and oral mucositis and alopecia [27]. However, this drug is not cardiotoxic, allowing its use in patients with cardiac impairment. In this regard, we have previously described one patient with *NPM1*-mutated AML unfit for intensive chemotherapy because of concomitant heart failure and refractory to 5-azacytidine, successfully treated with single-agent dactinomycin [24]. Here, we report the results of a phase 2 pilot clinical trial of single-agent dactinomycin in a cohort of 10 patients with relapsed or refractory (r/r) *NPM1*-mutated AML (EudraCT n. 2014-000693-18). Additionally, we define for the first time the pharmacokinetics of dactinomycin in adult AML patients and provide insights into the pathways involved in the response to this drug in patients.

## Methods

### Clinical study design

**Fig. 1:**
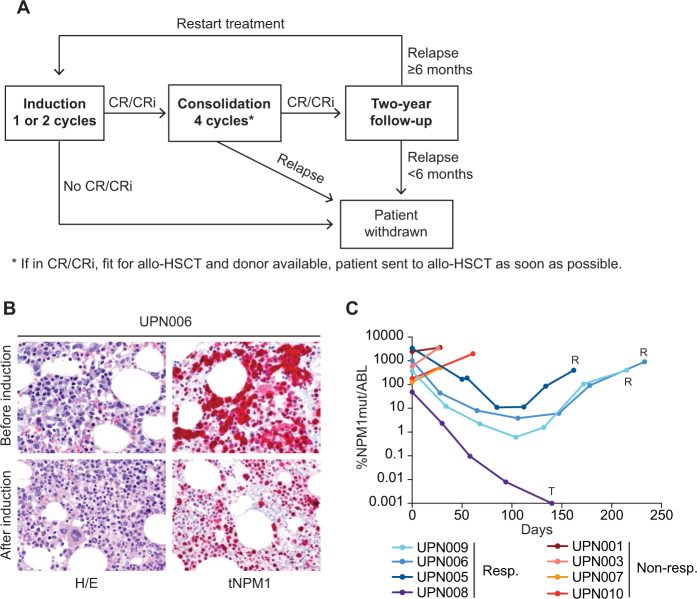
Dactinomycin can induce CR/CRi in *NPM1*-mutated AML patients. **A** Schematic representation of the study protocol. **B** Bone marrow biopsies of patient UPN006 before and after induction. Left panels are stained with hematoxylin and eosin (H/E), while right panels are stained with an anti-NPM1 (tNPM1), which recognizes both mutant and wild-type NPM1 (APAAP immunostaining, original magnification ×400). **C** Monitoring of NPM1mut transcripts by reverse-transcriptase real-time quantitative PCR in 8 out of 10 patients included in the protocol. Data are expressed as percentage of NPM1mut/ABL transcripts. Allo-HSCT, allogeneic hematopoietic stem cell transplant; R relapse, T transplant, Resp. responders, Non-resp. nonresponders.

The study was a single-center, single-arm, open-label, phase 2 pilot study to evaluate the safety and efficacy of dactinomycin in *NPM1*-mutated AML patients with hematological relapse or refractory disease. Relapse was established by morphological and immunohistochemical analysis (evidence of cytoplasmic NPM1). The protocol flow-chart is shown in Fig. 1A (for more details see Supplemental Materials and Protocol Synopsis). The protocol was approved by the Umbria Ethics Committee and the Italian Drug Agency and registered at the European Union Clinical Trial Register (clinicaltrialsregister.eu EudraCT n. 2014-000693-18). All patients signed a written informed consent. Dactinomycin was administered at 15 µg/kg/day for 5 consecutive days, which is the highest dose accepted according to the AHFS Drug Information [27]. Our decision to use slightly higher doses than those employed in gestational trophoblastic tumor (i.e., 10–12.5 µg/Kg/day for 5 days) [28] was based on the presumed higher aggressiveness of r/r *NPM1*-mutated AML. Administration of dactinomycin for 5 consecutive days followed by an interval of 2–4 weeks (depending on hematological and extra-hematological toxicity) defined one cycle of therapy.

Response was evaluated after one or two cycles. Complete response (CR) was defined as ≤5% BM leukemic cells with normalization of blood counts (neutrophils > 1.5 × 10^9^/L, hemoglobin ≥10 g/dL, platelets > 100 × 10^9^/L). CR with incomplete marrow recovery (CRi) was defined as ≤5% BM leukemic cells with incomplete recovery of blood counts. Patients not achieving CR or CRi (CR/CRi) after cycle 1 received a second induction cycle, unless progressive disease occurred. Patients not achieving CR/CRi after two cycles were withdrawn from the study. Patients achieving CR/CRi were allowed to receive a maximum of six cycles. Since in the original protocol criteria for response and relapse differed from those recommended by the European LeukemiaNet 2017 (ELN) [12], response and relapse rates were also calculated applying the ELN criteria, with absolutely no difference in the outcomes. Adverse events were graded according to the National Cancer Institute Common Terminology Criteria for Adverse Events, version 4.03.

### Clinical study statistical analysis

A Simon’s minimax two-stage design was adopted. We calculated a sample size that would be sufficient to accept the alternative hypothesis (CR/CRi rate after one or two induction cycles, ≥45%) and reject the null hypothesis (CR/CRi rate after one or two induction cycles, ≤10%), at an alpha level of 0.05 and a beta level of 0.2. Enrollment was closed in February 2016, when the prespecified number of patients (*n* = 10) had been enrolled. The study drug was to be considered worth of further investigation if CR/CRi was obtained in at least three patients.

Comparisons between multiple groups was done applying the Dunnett’s multiple comparison test with an alpa level of 0.05.

### Pharmacokinetics studies

Blood samples for quantification of dactinomycin concentrations were collected in EDTA tubes prior to drug administration and at 15, 30, 60, and 240 min post-administration. Plasma was obtained from whole blood by centrifugation at 1200 × *g* for 10 min at 4 °C and stored at −80 °C prior to shipment to the Newcastle University Centre for Cancer, UK for analysis. Dactinomycin levels were quantified using a fully validated liquid chromatography-mass spectrometry assay, with a limit of quantification of 0.25 ng/mL, as previously described [29, 30]. Briefly, extraction of clinical samples was carried out with acetonitrile and analysis performed on an API 4000 LC/MS/MS (AB SCIEX) using an internal standard of 7-aminoActD. The method has been demonstrated to exhibit good reproducibility over a calibration curve range of 0.25–100 ng/mL, with intra- and interassay precision and accuracy CVs and relative errors of <15%.

### In vitro experiments

Cell lines were purchased from the Leibniz Institute-DSMZ (Braunschweig, Germany) except for IMS-M2, which was kindly provided by Dr. Daniel G. Tenen (National University of Singapore, Singapore). Cells were cultured in RPMI (IMS-M2, HNT-34) or alfa-MEM (OCI-AML3, OCI-AML2) supplemented with 10% FBS, 1% penicillin–streptomycin, and 1% glutamine. Cells from a *NPM1*-mutated AML patient-derived xenograft model (PDX2) [31] and primary patient samples were kept in IMDM supplemented with 10% FBS, 1% penicillin–streptomycin, and 1% glutamine. Cells were exposed to dactinomycin at concentrations and time durations, as indicated. Cell viability was studied by flow cytometry with Annexin V and 7-AAD. Aliquots of treated cells were lysed for western blot analysis and aliquots were cytospun for immunofluorescence. Number of biological replicates is reported in figure legends.

### Engineering of isogenic cell lines

OCI-AML2 cells expressing doxycycline-inducible mutant NPM1 (NPM1mut) were engineered through lentiviral infection. pLVX-EF1a-Tet3G was used as regulator vector and pLVX-TRE3G-ZsGreen1 was used as response vector containing NPM1mut. Fresh doxycycline (100 ng/ml) was used for NPM1mut induction. Cells were either left untreated or treated with dactinomycin, as indicated.

### In vivo studies

BM aspiration was performed before starting (baseline) and 4 h following the first (day 1) and/or the fourth (day 4) administration of dactinomycin. Aliquots of BM mononucleated cells were used both for western blot analysis and cytospin for immunofluorescence.

### Targeted DNA sequencing

Genomic DNA was extracted from BM samples of 9/10 patients, prior to dactinomycin administration, using standard methods and subjected to molecularly-barcoded targeted sequencing of 40 myeloid genes (QIAseq Targeted DNA Custom Panel CDHS-13640Z-1017–QIAGEN). Targeted sequencing was also performed following one or two cycles of dactinomycin in the BM of 6/10 patients, including 4/4 patients who achieved CR/CRi, and was used to facilitate variant calling at diagnosis (see Supplemental Materials). Libraries generated according to the manufacturer’s instructions were sequenced on an Illumina MiSeq instrument, using MiSeq Reagent Kit v3 and V2.

## Results

### Single-agent dactinomycin demonstrates clinical activity in r/r *NPM1*-mutated AML

Between June 2014 and February 2016, 10 patients aged between 53 and 75 years (median 67) with r/r *NPM1*-mutated AML, who fulfilled all inclusion criteria, were enrolled onto the study. Patient characteristics are summarized in Table 1. Of 10 patients included, eight had relapsed disease (1–4 previous lines of treatment), and two had refractory disease progressing upon either 5-azacytidine or ‘7 + 3’ (Table 1). One patient (UPN004), who was colonized by a multiresistant *Klebsiella pneumoniae*, died early during induction 1 due to a septic shock caused by the same microorganism and was not considered evaluable for toxicity or response rate.

**Table 1:** Characteristics of R/R *NPM1*-mutated AML patients enrolled in the trial.

Patient	Age	Disease status	Previous therapy	*FLT3*	Response	Response duration
UPN001	66	Relapsed	CHT 2 lines	*FLT3*-ITD^homo^	PD	—
UPN002	66	Relapsed	CHT 2 lines	*FLT3*-ITD^homo^	PD	—
UPN003	75	Relapsed	CHT 1 line	*FLT3*-ITD^homo^	PD	—
UPN004	73	Relapsed	CHT 1 line	WT	Early Death	—
UPN005	71	Relapsed	CHT 4 lines	WT	CR (after 2 cycles)	3 months
UPN006	74	Refractory	HMA	WT	CR (after 1 cycle)	7 months
UPN007	53	Relapsed	HMA	WT	PD^a^	—
UPN008	63	Relapsed	CHT/AuSCT	WT	CRi (after 1 cycle)	4.7 years^b^
UPN009	66	Relapsed	CHT 1 line	*FLT3*-ITD	CR (after 2 cycles)	5 months
UPN010	67	Refractory	CHT 1 line	WT	PD^a^	—

*WT* wild-type, *CHT* cytotoxic chemotherapy, *PD* progressive disease, *HMA* hypomethylating agent, *AuSCT* autologous peripheral blood stem cell transplantation, *CR* complete remission, *CRi* complete remission with incomplete marrow recovery.

^a^Resistant to a further intensive chemotherapy regimen (UPN007: ‘7 + 3; UPN010: FLAI).

^b^Patient still alive in CR after HSCT.

Four out of the nine evaluable patients achieved CR/CRi after either one (UPN006, UPN008) or two (UPN005 and UPN009) induction cycles (Table 1). Morphologic CR was confirmed by immunohistochemistry [9], demonstrating the absence of cells with cytoplasmic NPM1 (Fig. 1B). A significant drop of *NPM1* mutant transcript copies (median 2.36 log, range 2.1–2.7) was observed by quantitative PCR analysis after two cycles in patients that achieved CR/CRi (Fig. 1C). Nevertheless, a temporary disappearance of leukemic cells from the PB was documented in all nine evaluable patients.

Patient UPN008 underwent haploidentical HSCT in CR after four cycles of dactinomycin and is still alive 4.7 years after transplant, with no detectable MRD measured by RT-qPCR. The other three patients who obtained CR relapsed after 3 (UPN005), 5 (UPN009), and 7 (UPN006) months. In all cases, hematological relapse was anticipated by increasing *NPM1* mutant transcripts (Fig. 1C) and detection of cytoplasmic NPM1 in BM blasts by immunohistochemistry (not shown).

Grade 4 anemia, thrombocytopenia and neutropenia consistently occurred during induction cycles, most likely due to leukemic BM infiltration. Grade ≥3 hematologic toxicities were also observed during consolidation cycles but were usually manageable in an outpatient setting. In fact, patients undergoing consolidation required only an average of <1 red cell unit and <1 platelet unit per cycle (average 0.91 unit/cycle for both red cell and platelet units; range 0–5 for red cell units, and 0–2 for platelet units). Interestingly, in the four patients who underwent at least two consolidation cycles, platelet nadir occurred at a median time of 11 days in both consolidation cycle 1 and consolidation cycle 2 (range 11–22 for consolidation 1 and 9–12 for consolidation 2), while neutrophil nadir occurred later at a median time of 17.5 days in consolidation 1 and 18 days in consolidation 2 (range 17–25 for consolidation 1 and 15–20 for consolidation 2).

Recurrent grade ≥3 extrahematologic toxicities were limited to oral mucositis, febrile neutropenia and sepsis (Table S1). Sepsis was reported only during the first cycle. Oral mucositis was transient but particularly unpleasant for patients, especially during the first cycle.

*FLT3* mutations (*FLT3*-ITD) were identified in 4/10 patients (Tables 1 and 2). In 3 of them (UPN001, 002, and 003) *FLT3*-ITD was found at a variant allele frequency >0.5, indicating homozygosity (*FLT3*-ITD^homo^). Notably, no patient carrying *FLT3*-ITD^homo^ achieved CR/CRi. To further establish the mutational profiles of patients enrolled in the study, we performed targeted DNA sequencing of 40 genes recurrently mutated in myeloid neoplasms (Table S2) on the BM sampled at the time of enrollment. Although the study was not powered to find association between specific mutations and response to treatment, 3/4 patients that achieved CR/CRi carried concomitant *IDH1* or *IDH2* mutations.

**Table 2:** DNA variants detected in patients at the time of enrollment in the study.

Patient	% Blast^a^	*FLT3*	Gene	VAF (%)	HGVS protein sequence	CR/CRi
UPN001	40–50	ITD^b^	N.A.	N.A.	—	No
UPN002	80	ITD^b^	*NPM1*	38.94	p.Trp288CysfsTer12	No
			*DNMT3A*	48.45	p.Arg882His	
UPN003	40	ITD^b^	*NPM1*	21	p.Trp288CysfsTer12	No
			*DNMT3A*	30.15	p.Arg882His	
			*RAD21*	26.31	p.Arg586Ter	
UPN004	80–90	WT	*NPM1*	39.21	p.Trp288CysfsTer12	N.E.
			*DNMT3A*	45.41	p.Arg882His	
			*IDH1*	43.25	p.Arg132His	
UPN005	80–90	WT	*NPM1*	35.54	p.Trp288CysfsTer12	Yes
			*SRSF2*	47.75	p.Pro95Arg	
			*IDH2*	45.87	p.Arg140Gln	
			*CEBPA*	41.57	p.Asp80GlyfsTer28	
UPN006	40–50	WT	*NPM1*	34.98	p.Trp288CysfsTer12	Yes
			*DNMT3A*	42.79	p.Phe732Leu	
			*RAD21*	33.24	p.Ile499LysfsTer5	
			*PTPN11*	10.34	p.Gly60Ala	
UPN007	40–50	WT	*NPM1*	17.06	p.Trp288CysfsTer12	No
			*GATA2*	18.31	p.His380Gln	
			*GATA2*	1.3	p.Arg362Gly	
UPN008	15–20	WT	*NPM1*	/^c^	/^c^	Yes
			*IDH1*	16.96	p.Arg132Ser	
			*TET2*	4.05	p.His1219Arg	
UPN009	20	ITD	*NPM1*	5.62^c^	p.Trp288CysfsTer12	Yes
			*IDH1*	4.66	p.Arg132His	
UPN010	20–25	WT	*NPM1*	/^c^	/^c^	No
			*BCORL1*	2.05	p.Leu647Val	
			*IDH1*	1.26	p.Arg132His	

*N.A*. not available, *N.E*. not evaluable, *VAF* variant allele frequency.

^a^Blast percentage on BM biopsy or aspirate, prior to treatment.

^b^FLT3-ITD VAF > 50%.

^c^Samples with marked peripheral blood contamination;/Variant frequency below the threshold for calling. NPM1 mutations were confirmed in all patients by immunohistochemistry and by conventional molecular techniques.

These results demonstrate that single-agent dactinomycin 15 µg/kg/day for 5 consecutive days can induce CR in relapsed/refractory *NPM1*-mutated AML. Oral mucositis was the main limiting toxicity at this dosage.

### Pharmacokinetics of dactinomycin

**Fig. 2:**
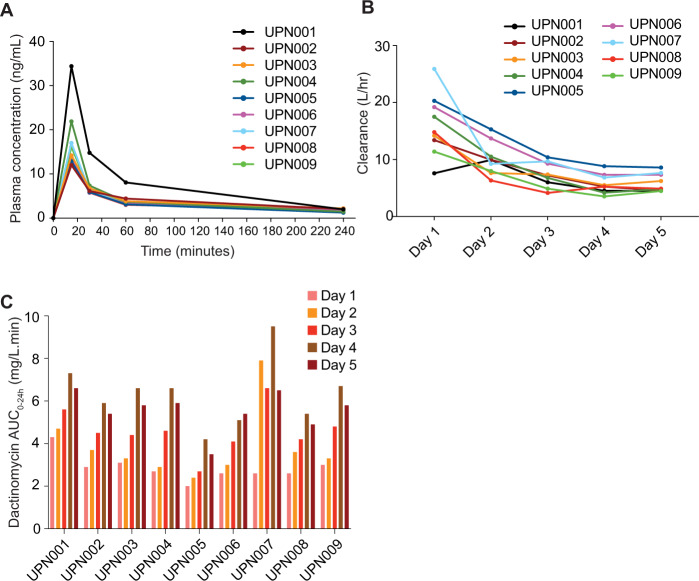
Pharmacokinetics of dactinomycin. **A** Plasma concentrations of dactinomycin following the first administration in each patient. **B** Dactinomycin clearance during the 5 days of treatment (cycle 1) in each patient. **C** Dactinomycin AUC_0–24h_ during the 5 days of treatment (cycle 1) in each patient.

To establish the pharmacokinetics of dactinomycin in adult patients with *NPM1*-mutated AML, we serially determined drug plasma concentrations by mass spectrometry [30] in nine patients. As previously reported in pediatric populations [29], on day 1 C_max_ values ranged between 12 and 34.4 ng/ml (Fig. 2A), clearance values ranged between 7.6 and 25.9 L/h (Fig. 2B) and AUC_0-24h_ ranged between 2.0 and 4.3 mg/L.min (Fig. 2C) across the nine patients. However, we registered a consistent and progressive reduction of the drug clearance over the following 4 days of treatment (Fig. 2B). This resulted in higher exposures to dactinomycin on day 5 as compared to day 1, with AUC_0-24h_ values observed ranging from 3.5–6.6 mg/L.min at day 5 (Fig. 2C). Of note, UPN007 who had mild renal impairment (estimated glomerular filtration rate 51 ml/min/1.73m [2]), showed a marked reduction in drug clearance beginning at day 2 and the highest AUC_0–24h_ values (Fig. 2B, C). These findings suggest that the dose or the treatment duration may need to be reduced in patients with renal impairment.

Neither C_max_ nor AUC_0–24h_ values correlated with remission induction. However, we observed a correlation between the severity of oral mucositis and dactinomycin plasma concentrations. In fact, grade 3 mucositis was observed in UPN001, 002, 003 and 009, who reported AUC_0–24h_ values higher than other patients (except UPN007), suggesting that decreasing the dose of dactinomycin may reduce the grade of mucositis while retaining antileukemic activity.

### Pulsed doses of dactinomycin induce nucleolar stress in vitro and in vivo

To investigate whether the drug activity correlates with the nucleolar stress response, we exposed *NPM1*-mutated leukemic cells to pulsed doses dactinomycin in vitro. Based on our PK studies and previously published data [17–19], we incubated AML cells with dactinomycin concentrations ranging between 0.005 and 0.1 μg/ml, for 1–6 h. The drug was then washed out and AML cells were placed in fresh medium. When appropriate, pulsed treatment was repeated for up to 5 consecutive days, reproducing the schedule adopted in patients.

**Fig. 3:**
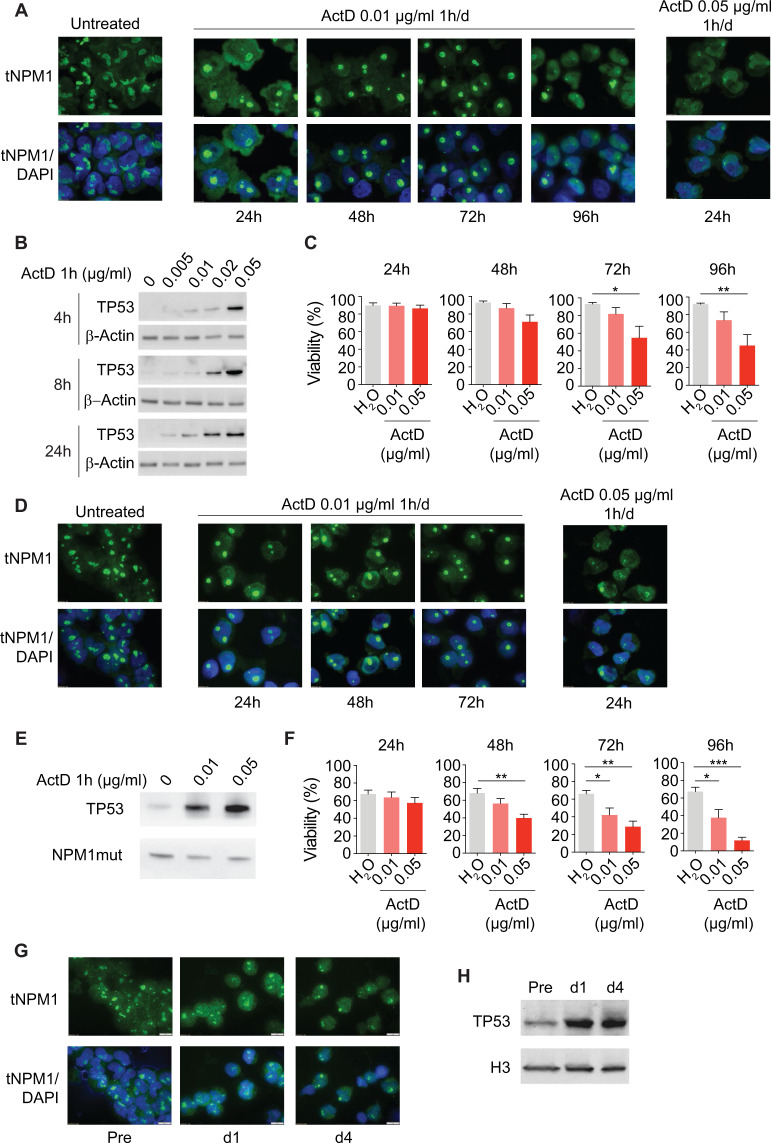
Dactinomycin induces nucleolar stress in *NPM1*-mutated AML cells in vitro and in patients. **A** Immunofluorescence of OCI-AML3 cells treated with dactinomycin at the indicated concentrations for 1 h/day for a maximum of 4 consecutive days, stained with an anti-NPM1, which recognizes both mutant and wild-type NPM1 (tNPM1). **B** Western blot analysis of TP53 and β-actin in OCI-AML3 cells treated with dactinomycin at the indicated concentration for 1 h. **C** Viability of OCI-AML3 cells treated with dactinomycin at the indicated concentration for 1 h/day for 4 consecutive days and studied by flow cytometry with 7-AAD and Annexin V. Results are expressed as percentage of 7-AAD/Annexin V double-negative cells (*n* = 4, Dunnett’s multiple comparison). **D** Immunofluorescence of PDX2 cells treated with dactinomycin at the indicated concentrations for 1 h/day for a maximum of 4 consecutive days, stained with the anti-tNPM1. **E** Western blot analysis of TP53 in PDX2 cells treated with dactinomycin at the indicated concentration for 1 h. **F** Viability of PDX2 cells treated with dactinomycin at the indicated concentration for 1 h/day for 4 consecutive days and studied by flow cytometry with 7-AAD and Annexin V. Results are expressed as percentage of 7-AAD/Annexin V double-negative cells (*n* = 4, Dunnett’s multiple comparison). **G** Immunofluorescence of cells isolated from the bone marrow of patient UPN002 before starting the treatment (Pre), 4 h following the first administration (d1), and 4 h after the fourth administration (d4). Cells were stained with the anti-tNPM1. **H** Western blot analysis of TP53 in cells isolated from the bone marrow of patient UPN002 before starting the treatment (Pre), 4 h following the first administration (d1), and 4 h after the fourth administration (d4). ActD, dactinomycin. *p < 0.05, **p < 0.01, ***p < 0.001. Error bars indicate s.e.m. Scale bar: 10 μm.

We first tested our strategy in the *NPM1*-mutated AML cell line OCI-AML3. One-hour/day exposure to dactinomycin induced progressive nucleolar changes (Fig. 3A), indicative of nucleolar stress [32, 33]. Specifically, NPM1 diffused from the nucleoli to the nucleoplasm, with concomitant loss of the irregular nucleolar shape and gain of a more spheroidal shape (Fig. 3A). Changes in NPM1 distribution became more evident over time, with a nearly complete nucleoplasmic diffusion at 96 h (Fig. 3A). No consistent changes in the distribution of cytoplasmic NPM1 were observed (not shown).

NPM1 can bind to and inhibit the ubiquitin-ligase HDM2, one of the key regulators of TP53 levels [34, 35]. Dactinomycin-induced NPM1 diffusion to the nucleoplasm is known to result in NPM1-HDM2 interaction and HDM2 inhibition, leading to increased TP53 levels [34, 35]. Indeed, changes in NPM1 nuclear distribution were associated with a progressive increase in TP53 (Fig. 3B), followed by dose-dependent cell death in OCI-AML3 cells exposed to dactinomycin (Fig. 3C). This response pattern was confirmed in PDX2 cells (Fig. 3D–F) as well as in a different *NPM1*-mutated AML cell line (IMS-M2) [36] (Fig. S1A and not shown).

To confirm that nucleolar stress response can be induced in primary AML cells, we applied the same strategy to treat fresh AML samples from two patients included in the study (UPN002 and 004) (Fig. S1B, S1C, S1D, S1E) and from the previously reported patient (Fig. S1F), demonstrating consistent NPM1 diffusion to the nucleoplasm coupled with increased TP53 levels.

Finally, to demonstrate that dactinomycin can elicit nucleolar stress in patients, we performed sequential BM aspiration at baseline, 4 h after the first administration (day 1) and 4 h following the fourth administration (day 4) in one patient (UPN002), confirming progressive changes in the nucleolar morphology, NPM1 nucleoplasmic diffusion and increased TP53 levels (Fig. 3G, H). Early TP53 increase (day 1) was also confirmed in the BM of a second patient (UPN004) (Fig. S1G).

Altogether, these data demonstrate that pulsed dactinomycin induces nucleolar stress in *NPM1*-mutated AML cells as early as 4 h both in vitro and in leukemic patients undergoing treatment.

### Differential nucleolar stress response in *NPM1*-mutated versus *NPM1* wild-type AML cells

**Fig. 4:**
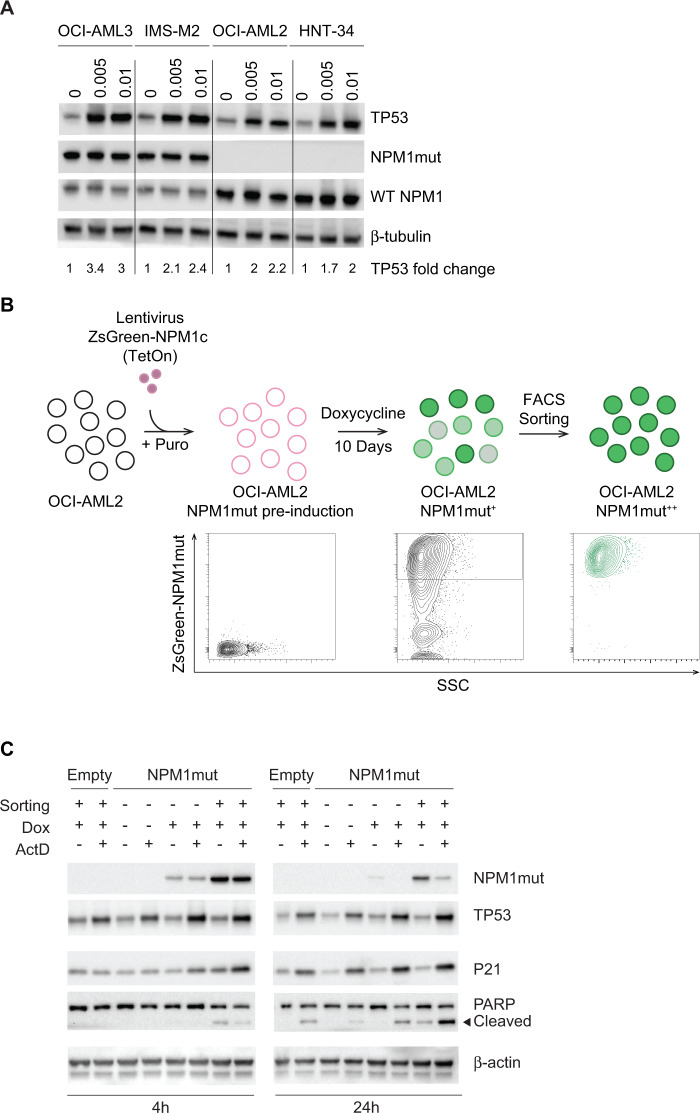
NPM1mut facilitates nucleolar stress response to dactinomycin. **A** Western blot analysis of TP53, NPM1mut and wild-type NPM1 (WT NPM1) in OCI-AML3, IMS-M2, OCI-AML2, and HNT-34 cells, treated with dactinomycin at the indicated concentration (μg/mL) for 6 h. Quantification of TP53 fold changes is reported. See also Fig. S2A. **B** Cartoon illustrating the design of the experiment performed on OCI-AML2 cells engineered to express doxycycline-inducible NPM1mut. **C** Western blot analysis of NPM1mut, TP53, P21, and PARP in engineered OCI-AML2 treated with dactinomycin 0.05 μg/mL for 1 h and harvested 3 and 23 h later. Dox doxycycline. ActD dactinomycin.

To explore whether *NPM1*-mutated cells may have lower thresholds for nucleolar stress response than *NPM1* wild-type cells, we exposed to dactinomycin OCI-AML3 and IMS-M2 cells as well as OCI-AML2 and HNT-34 cells (both *NPM1* wild-type) [37], all of which have detectable TP53 levels by western blot. In vitro exposure to 6-hour low-dose dactinomycin resulted in higher TP53 levels in *NPM1*-mutated cell lines, compared to *NPM1* wild-type cells (Figs. 4A and S2A).

As we could not discriminate whether higher TP53 levels in *NPM1*-mutated cells were secondary to factors other than just the presence of mutant NPM1, we engineered *NPM1* wild-type OCI-AML2 cells to express a doxycycline-inducible mutant NPM1 fused to the fluorescent protein ZsGreen1 (ZsGreen1-NPM1c) (Fig. 4B). Engineered cells were incubated with doxycycline for 10 days and then treated with pulsed dactinomycin either as bulk or following sorting for high ZsGreen1-NPM1c levels (Fig. 4B). OCI-AML2 cells transduced with a doxycycline-inducible ZsGreen1 empty vector and sorted for high ZsGreen1 levels were used as control. In vitro pulsed dactinomycin produced higher levels of TP53, its downstream P21 as well as cleaved poly(ADP-ribose) polymerase (PARP) in cells expressing mutant NPM1, compared to control. Furthermore, cells expressing the highest ZsGreen-NPM1c levels showed the strongest TP53 activation and apoptosis induction at 24 h (Fig. 4C). In conclusion, expression of NPM1 mutant protein in *NPM1* wild-type cells confers higher sensitivity to dactinomycin as measured by TP53 and P21 levels, which is proportional to NPM1mut expression levels.

Altogether, these results support the hypothesis that *NPM1*-mutated AML cells have lower thresholds for dactinomycin-induced nucleolar stress response. However, the mechanisms through which mutant NPM1 sensitizes AML cells to dactinomycin remain to be clarified.

## Discussion

Here, we report the results of the first phase 2 trial evaluating the safety and efficacy of dactinomycin in r/r *NPM1*-mutated AML. The primary endpoint of the study was met since CR/CRi was obtained in 4/9 evaluable patients. While three patients who initially responded to the drug relapsed within seven months, one patient who underwent allo-HSCT is still in molecular CR > 4 years later. This study follows the previous report of one *NPM1*-mutated AML patient unfit for intensive CHT due to congestive heart failure, who was successfully treated with single-agent dactinomycin at the dose of 12.5 μg/Kg/day for 5 days [24]. The patient remains in molecular CR, 6 years following treatment and is considered cured. Our results suggest that dactinomycin administered as single-agent in *NPM1*-mutated r/r patients can induce CR and can be successfully used to bridge patients to allo-HSCT, although these data need validation in a larger cohort.

PK studies conducted in these patients generated novel data demonstrating that dactinomycin clearance values decrease progressively across the 5 days of treatment. With the caveat of the limited number of patients, dactinomycin AUC correlated with toxicity, particularly mucositis, but not with CR/CRi. These data, along with our previous report [24], suggest that doses lower than 15 μg/Kg/day (e.g., 10–12.5 μg/Kg/day for 5 days) could be equally effective but associated with reduced toxicity. In this regard, a multicenter Phase 2 study to establish the efficacy of dactinomycin 12.5 μg/Kg/day for 5 days in *NPM1*-mutated AML patients unfit or relapsed after hypomethylating agents (HMAs) is currently ongoing (EudraCT n.2014-003490-41). Recently, off-label dactinomycin 12.5 μg/Kg/day for 5 days has been tested in a heterogeneous group of 26 adult patients with *NPM1*-mutated AML (mostly relapsed/refractory), with a reported CR rate of 18% [38]. However, due to the high heterogeneity and the retrospective fashion of the study, no statistical conclusion on the efficacy of this dose could be drawn.

Combinations of HMAs and venetoclax have recently demonstrated high efficacy in unfit older patients with *NPM1*-mutated AML, although patients eventually relapse [16, 39]. Older unfit *NPM1*-mutated AML patients relapsing after venetoclax-based regimens represent a medical need and dactinomycin could be an inexpensive therapeutic option in this setting.

The mechanisms through which dactinomycin exert its antileukemic activity is unclear. The finding that *NPM1*-mutated cells seem to have lower thresholds for nucleolar stress response in vitro supports our previous hypothesis that higher levels of NPM1 in the nucleoplasm may help trigger dactinomycin-mediated nucleolar stress response, contributing to the high activity of the drug in *NPM1*-mutated AML [24]. Since RNA Pol I inhibition by dactinomycin reduces rRNA concentration in the nucleoli resulting in loss of NPM1 phase separation from the nucleoplasm [40], we speculate that in *NPM1*-mutated cells, where NPM1 already tends to be delocalized to the nucleoplasm, dactinomycin may exacerbate such delocalization, stimulating nucleolar stress response. However, other mechanisms may contribute to the antileukemic activity of dactinomycin in *NPM1*-mutated AML (H. de Thé, unpublished results).

The impact of co-occurring mutations on the sensitivity of *NPM1*-mutated cells to dactinomycin could not be established due to the limited sample size. However, three of the four patients who achieved CR/CRi carried mutations of either *IDH1* or *IDH2*. Interestingly, the previously reported *NPM1*-mutated patient cured by single-agent dactinomycin also carried an *IDH2* mutation. These data suggest that the combination of *IDH* and *NPM1* mutations may confer sensitivity to dactinomycin, but this hypothesis needs further validation. Interestingly, *IDH*-mutated hematopoietic cells display altered DNA-damage response with consequent accumulation of DNA damage [41, 42]. We speculate that this background may itself facilitate a TP53-mediated response and dactinomycin-induced nucleolar stress would exacerbate the activation of TP53 downstream signals. This hypothesis, which needs to be validated and could be partially independent from nucleolar stress, may also explain the promising sensitivity of *IDH/NPM1* double-mutated AML to other compounds (e.g., venetoclax).

Other drugs causing nucleolar stress through inhibition of ribosome biogenesis [43] are also worthy of investigation in *NPM1*-mutated AML. For instance, anthracyclines, such as daunorubicin, have been demonstrated to induce nucleolar stress response coupled with NPM1 relocation to the nucleoplasm in vitro [22], similar to dactinomycin. Intriguingly, *NPM1*-mutated AML shows excellent response rates to anthracycline-containing regimens [11].

In conclusion, it remains to be established whether: (i) dactinomycin could be used at lower doses with lower toxicity and similar efficacy; (ii) dactinomycin may represent an option for *NPM1-*mutated AML patients unfit or older than 75 years refractory to or relapsed after venetoclax-based regimens; and (iii) combination of dactinomycin with newer agents (e.g., HMAs, venetoclax, FLT3 inhibitors) may yield better results, particularly in *NPM1*-mutated patients carrying *FLT3*-ITD. Furthermore, deeper understanding of the mechanisms behind nucleolar stress response and the apparent higher sensitivity of *NPM1*-mutated cells to dactinomycin will help optimize new therapeutic approaches in this setting.

## Supplementary information

Supplemental materials

Protocol Synopsis
