# Supplementary material for: Dactinomycin induces complete remission associated with nucleolar stress response in relapsed/refractory NPM1-mutated AML
Source: Leukemia. 2021 Mar 2;35(9):2552–62. doi: 10.1038/s41375-021-01192-7 (PMC8410589; doi:10.1038/s41375-021-01192-7)
Supplement: Supplementary file 1 — Supplemental materials [file 41375_2021_1192_MOESM1_ESM.doc]

**SUPPLEMENTARY FIGURES AND TABLES**

**Figure S1**

**Figure S1. Dactinomycin induces nucleolar stress response in vitro and in vivo in *NPM1*-mutated AML cells.** (A) Western-blot analysis of TP53 in IMS-M2 cells treated with dactinomycin at the indicated concentration for 1 hour and harvested at the indicated timepoint. (B) Immunofluorescence of primary AML cells from UPN002 treated *in vitro* with dactinomycin at the indicated concentrations for 4 hours, stained with an anti-NPM1 which recognizes both mutant and wild-type NPM1 (tNPM1). (C) Western-blot analysis of TP53 in primary cells from UPN002 treated *in vitro* with dactinomycin at the indicated concentration for 1 hour and harvested at the indicated timepoint. (D) Immunofluorescence of primary AML cells from UPN004 treated *in vitro* with dactinomycin at the indicated concentrations for 4 hours, stained with anti-tNPM1. (E) Western-blot analysis of TP53 in primary cells from UPN004 treated *in vitro* with dactinomycin at the indicated concentration for 1 hour and harvested at the indicated timepoint. (F) Western-blot analysis of TP53 in primary cells from the previously reported patient treated *in vitro* with dactinomycin at the indicated concentration for 1 hour and harvested at the indicated timepoint. (G) Western-blot analysis of TP53 of AML cells harvested from the BM of in UPN004 at baseline (Pre) and 4 hours following the first administration of dactinomycin (d1). Scale bar: 10 μm. ActD: dactinomycin.

**­**

**Figure S2. NPM1mut facilitates stress response to dactinomycin.** (A) Western blot analysis of TP53 in OCI-AML3, IMS-M2, OCI-AML2 and HNT-34 cells, treated with dactinomycin at the indicated concentration (μg/mL) for 6 hours. Quantification of TP53 fold changes is reported. See also Figure 4A. Independent replicate.

**Table S1. Non-hematologic toxicities of grade ≥3.**

| **Event^** | **Induction 1 (n=9)** | **Induction 2 (n=4)** | **Consolidation (n=4)** |
| --- | --- | --- | --- |
| Febrile neutropenia | 4 | 3 | 3^*^ |
| Oral mucositis | 4 | 1 | 2 |
| Sepsis | 3 | 0 | 0 |
| Urinary tract infection | 1 | 1 | 1 |
| Any increased aminotransferase | 0 | 1 | 0 |
| Cutaneous Ulcers | 1 | 0 | 1 |
| QTc prolongation | 0 | 1 | 0 |
| Hyponatremia | 0 | 0 | 1 |
| Viremia | 1** | 0 | 0 |

n=number of patients

^ Reported toxicities were all of grade 3 except for one grade 4 sepsis.

* Maximum 3 days

** Herpes Simplex Virus 1

**­**

**Table S2. Complete list of genes and exons included in the sequencing panel.**

| **Gene** | **Target (exon)** | **Gene** | **Target (exon)** | **Gene** | **Target (exon)** | **Gene** | **Target (exon)** |
| --- | --- | --- | --- | --- | --- | --- | --- |
| ***ASXL1*** | 12 | ***GATA1*** | 2 | ***MPL*** | 10 | ***SF3B1*** | 13-16 |
| ***BCOR*** | All exons | ***GATA2*** | 4-6 | ***NPM1*** | 12 | ***SMC1A*** | 2, 11, 16, 17 |
| ***BCORL1*** | All exons | ***HRAS*** | 2,3 | ***NRAS*** | 2, 3 | ***SMC3*** | 10, 13, 19, 23, 25, 28 |
| ***BRAF*** | 15 | ***IDH1*** | 4 | ***PDGFRA*** | 12, 14, 18 | ***SRSF2*** | 1 |
| ***CALR*** | 9 | ***IDH2*** | 4 | ***PHF6*** | All exons | ***STAG2*** | All exons |
| ***CEBPA*** | All exons | ***JAK2*** | 12,14 | ***PTEN*** | 5, 7 | ***TET2*** | 3-11 |
| ***CSF3R*** | 14-18 | ***JAK3*** | 13 | ***PTPN11*** | 3, 13 | ***TP53*** | 2-11 |
| ***DNMT3A*** | All exons | ***KIT*** | 2, 8-11, 13, 17 | ***RAD21*** | All exons | ***U2AF1*** | 2, 6 |
| ***EZH2*** | All exons | ***KRAS*** | 2, 3 | ***RUNX1*** | All exons | ***ZRSR2*** | All exons |
| ***FLT3*** | 14, 15, 20 | ***MLL*** | 1, 3, 5-8, 27 | ***SETBP1*** | 4 (partial) | ***PPM1D*** | 5-6 |

**SUPPLEMENTARY METHODS**

**Patients**

Ten patients were enrolled in the study. Patients meeting the following criteria were included: a) patients aged 18-60 years with *NPM1*-mutated AML whose disease was refractory to or relapsed after at least two different lines of therapy; b) patients aged ≥60 years with *NPM1*-mutated AML whose disease was refractory to or relapsed after one line of therapy. Detailed inclusion and exclusion criteria are provided in the protocol synopsis.

**Study design**

The study was designed as single-center, open label, phase 2, single arm study. Dactinomycin was administered intravenously at 15 µg/Kg/die for 5 consecutive days, at approximately the same time each day. Administration of dactinomycin for 5 consecutive days followed by an interval of 2-4 weeks (depending on hematological and extra-hematological toxicity) defined one cycle of therapy. Patients received a maximum of six cycles of dactinomycin, unless eligible for allotransplant or withdrawn from the study. Specifically, patients in complete remission (CR or CRi) after the first cycle of dactinomycin (induction 1) continued on treatment for additional four cycles (consolidation 1-4) for a total of five cycles. Patients that did not obtain complete remission (CR or CRi) after induction 1 received a second induction cycle (induction 2), unless progressive disease was established. Patients in CR after induction 2 received four consolidation cycles with dactinomycin for a total of six cycles. Patients not achieving CR after two induction cycles or demonstrating disease relapse or progression at any time after the 2 induction cycles were withdrawn from the study. Further details are provided in the protocol synopsis.

**Study assessments**

Adverse events were graded according to the National Cancer Institute Common Terminology Criteria for Adverse Events, version 4.03. Assessment of response based on blood counts, bone marrow (BM) biopsy and aspirate was performed before starting each cycle of therapy, then monthly between month 1 and 3 of follow-up and then every three months between month 4 and 24 of follow-up.

Response was evaluated after one or two cycles. Complete response (CR) was defined as ≤5% BM leukemic cells with normalization of blood counts (neutrophils >1.5x10^9^/L, hemoglobin ≥10 g/dL, platelets >100x10^9^/L). CR with incomplete marrow recovery (CRi) was defined as ≤5% BM leukemic cells with incomplete recovery of blood counts. Patients not achieving CR or CRi (CR/CRi) after cycle 1 received a second induction cycle, unless progressive disease occurred. Patients not achieving CR/CRi after two cycles were withdrawn from the study. Patients achieving CR/CRi were allowed to receive a maximum of 6 cycles. Partial response (PR) was defined as decrease of bone marrow blast percentage to 5-25% and decrease of pretreatment bone marrow blast percentage by at least 50%. No response was defined as the presence of ≥20% leukemic cells in the BM after two induction cycles. In patients achieving a CR or CRi after induction. Relapse was defined as the reappearance of at least 5% blasts in BM, irrespectively of neutrophil count, hemoglobin level and platelet count. Since in the original protocol criteria for response and relapse differed from those recommended by the European LeukemiaNet 2017 (ELN)^1^, response and relapse rates were also calculated applying the ELN criteria, with absolutely no difference in the outcomes.

**Clinical study statistical analysis**

A Simon’s minimax two-stage design was adopted. We calculated a sample size that would be sufficient to accept the alternative hypothesis (CR rate after one or two induction cycles, ≥45%) and reject the null hypothesis (CR rate after one or two induction cycles, ≤10%), at an alpha level of 0.05 and a beta level of 0.2. Enrollment was closed in February 2016, when the pre-specified number of patients (n=10) had been enrolled.

**Immunohistochemical Analysis.**

Histologic and immunohistochemical analysis was performed on bone marrow biopsies paraffin-embedded samples, fixed in B5 (Bio-Optica) and processed as previously described^2^. Briefly, paraffin sections from fixed histological samples were subjected to antigen retrieval for 5 minutes at 85°C with Dako Target Retrieval Solution/High pH (Dako, cat. K8004) in Dako PT link (Dako, cat. PT101) and immunostained with primary antibodies. Antibody/antigen reaction was revealed using the Dako REAL LSAB+ kit detection system (Dako, cat. K5005). Sections were then counterstained in hematoxylin for 5 minutes. Cytoplasmic nucleophosmin was revealed using a mouse anti-NPM1 monoclonal antibody (Clone 376, produced by B.F.), recognizing both wild-type and mutant NPM1 (tNPM1)^2^. Immunopathology images were acquired with the 340/0.85 (40x) objective (Olympus U Plan Apo) of an Olympus B61 microscope equipped with an Olympus E330-ADU1.2xcamera, using the Olympus cell^B acquisition software, and processed with Adobe Photoshop 7.0 and Adobe Illustrator CC 2015.

**Cell lines, human samples and cell culture**

The human AML cell lines, OCI-AML3 and IMS-M2 (carrying *NPM1* mutation A), and OCI-AML2 and HNT-34 (with wild-type *NPM1*) were previously reported^3-6^. The primary *NPM1*-mutated AML subcutaneous xenograft model (PDX2) was also previously reported^7^. Primary AML cells were obtained upon written informed consent from patients either at baseline or upon treatment with dactinomycin within the clinical trial, as indicated. The study was conducted according to the Declaration of Helsinki and approved by the regional ethics committee (CEAS Umbria). Human primary AML cells (UPN002 and 004) were isolated by density gradient centrifugation of either peripheral blood or BM. AML was defined as *NPM1*-mutated or *NPM1*-wild type based on cytoplasmic or nuclear expression of nucleophosmin at immunohistochemistry, which is predictive of *NPM1* mutational status. Expression of the NPM1 mutant protein was documented at western blot analysis with anti-NPM1 mutant specific antibodies, as previously reported ^8^.

Cells were cultured in RPMI (IMS-M2 and HNT34) or alfa-MEM (OCI-AML3 and OCI-AML2) supplemented with 10% FBS, 1% penicillin-streptomycin and 1% glutamine at concentrations ranging between ~2.5 x 10^6^ and ~1 x 10^6^ cells/ml. PDX2 cells and primary patient samples were kept in IMDM supplemented with 10% FBS, 1% penicillin-streptomycin and 1% glutamine at a concentration of ~2 x 10^6^ cells/ml.

**Engineering of isogenic cell lines**

OCI/AML2 cell line expressing NPM1 mutant A (NPM1mut) was obtained by lentiviral infection. Particularly, pLVX-EF1a-Tet3G was used as regulator vector and pLVX-TRE3G-ZsGreen1 was used as response vector containing NPM1c. OCI/AML2 double-transduced cells were maintained in RPMI 1640 medium supplemented with 10% of tetracycline free fetal bovine serum, 2 mM glutamine, 2 mM penicillin/streptomycin, and antibiotics (1 mg/ml of G418 and 1.5 µg/ml puromycin, used at 1/3 of the rates used for selection) to maintain the selective pressure. For pharmacological experiments, induction of *NPM1*mut transcription was obtained by doxycycline treatment according to the following treatment: i) 10 days before the experiment double-transduced cells were split in two group: “induced” and “not induced”. Fresh doxycycline at 100 ng/ml was maintained only in the ‘induced’ group. Cells were plated at 3.5 x 10^5^ cells/ml and either left untreated or treated with dactinomycin, as indicated.

**Drugs and cell treatment**

Dactinomycin was purchased from Sigma Aldrich (St Louis, MO, USA). For cell lines treatments, cells were plated as follows: 0.25 × 10^6^/ml for OCI-AML3, 0.35 × 10^6^/ml for OCI-AML2 and IMS-M2, 0.5 × 10^6^/ml for HNT-34. Dactinomycin was resuspended at 1 mg/ml in PBS. Further dilutions were performed in PBS as well. For pharmacological experiments, cells were exposed to dactinomycin at different concentrations for 1 to 6 hours, as indicated. Following exposure, drug was washed out by rinsing cells with PBS and cells were put back in fresh medium without drug for sampling at the indicated time points. When indicated, incubation with the drug was repeated at 24 hours intervals.

**Cell lysates preparation and Western Blot analysis**

Fresh cell pellets (0.2-0.5 × 10^6^/test for AML cell lines; 0.5-1 × 10^6^/test for primary AML cells) were dissolved directly in 30-60 ml of Laemmli sample buffer 1X (1,5 M Tris-HCL Ph 6.8, Glycerol, β-mercaptoethanol, SDS, Bromophenol blue) and boiled at 95°C for 5 minutes. Proteins were separated by SDS-polyacrylamide gel electrophoresis on 4-15% gradient gels (Biorad, Hercules, CA, USA), transferred onto nitrocellulose membranes (GE Healthcare, Piscataway, NJ, USA) and probed with specific primary antibodies followed by horseradish peroxidase-conjugated secondary antibodies (GE Healthcare). Polypeptides were visualized using enhanced chemoluminescence (ECL, GE Healthcare Lifesciences, or Luminata Crescendo, Millipore, Billerica, Massachusetts, USA) according to the manufacturer’s instructions. Bands were visualized using the Biorad Chemidoc system and images were processed using the image lab software (Biorad). Histone H3, β-Tubulin or β-actin expression levels were used as control for protein loading, as indicated. The band intensity of each target was quantified using ImageJ analysis software freely available at http://rsb.info.nih.gov/ij/ and normalized to loading control band intensity in each lane.

**Antibodies**

Primary antibodies used for western blot analyses were: mouse anti-human β-Tubulin (Clone TUB 2.1; dilution 1:1000) and mouse anti-human β-actin (Clone AC-15; dilution 1:5000) from Sigma Aldrich (St. Louis, MO, USA); rabbit anti-human Cleaved poly(ADP-ribose)polymerase (PARP) (Clone Asp 214; dilution 1:1000) and rabbit anti-human Histone H3 (Clone D1H2; dilution 1:1000) from Cell Signaling (Danvers, MA, USA). The mouse monoclonal antibody specifically recognizing wild-type NPM1 (Clone FC-61991; dilution 1:1000) was from Invitrogen (Carlsbad, CA, USA); the rabbit polyclonal anti-human mutated NPM1 (NPM1mut) was previously described ^8^. The primary antibody used for immunofluorescence was a mouse anti-human Nucleophosmin 1 (Human Protein Atlas Number HPA011384, diluition 1:1000) from Sigma Aldrich (St. Louis, MO, USA), recognizing both wild-type and mutant NPM1 (tNPM1).

**Viability by flow-cytometry**

Cell apoptosis was assessed using Annexin V APC-conjugated antibody and counterstained with 7-AAD for detection of necrotic cells (Becton Dickinson, Franklin Lakes, New Jersey, USA), according to manufacturer’s protocol. Data acquisition and analysis were done with a FACSCalibur flow cytometer using CellQuest software (Becton Dickinson, Franklin Lakes, New Jersey, USA). Viable cells were gated as AnnexinV/7-AAD double negative cells.

**Immunofluorescence**

For immunofluorescence analysis, either untreated or treated cells were washed and cytospun on slides, allowed to dry o/n and fixed with 4% paraformaldehyde for 10 minutes. After washing in PBS, cells were permeabilized with 0.1% Triton X-100 for 5 minutes, washed again and blocked with 1% BSA before incubation with the primary mouse monoclonal anti-human PML antibody, PG-M3 hybridoma supernatant (1:8 dilution in 1% BSA) at RT for 1 hour or o/n at 4°C in a humidified chamber. After washing, cells were incubated with secondary goat anti-mouse Alexa-Fluor 568 (red) IgG secondary fluoresce­­­­nt antibody (Molecular Probes by Life Technologies) for 30 minutes at RT in the dark. For simultaneous NPM1 detection, the rabbit anti-human NPM1 antibody (1:1000 dilution in 1% BSA) (Sigma Aldrich (St. Louis, MO, USA) and goat anti-rabbit Alexa-Fluor 488 (green) IgG secondary fluoresce­­­­nt antibody (Molecular Probes by Life Technologies) were used. Nuclei were stained with 4,6-DiAmidino-2-PhenylIndole (DAPI) in Prolong Gold mounting reagent (Molecular Probes by Life Technologies). Immunofluorescence images were collected at fluorescence microscope (Olympus, Shinjuku, Tokyo, Japan) and processed with CellSens Digital Imaging Software (Olympus).

**In vitro experiments statistical analysis**

All the experiments were performed at least in triplicates, as indicated, with technical repetition when possible. Results are expressed as mean ± standard error (SEM). Comparison between groups was performed applying the Dunnett’s multiple comparison following an ordinary one-way ANOVA.

**Targeted DNA sequencing**

Genomic DNA was extracted from BM samples of 9/10 patients prior to dactinomycin using standard methods and subjected to molecularly-barcoded targeted sequencing of 40 myeloid genes (QIAseq Targeted DNA Custom Panel CDHS-13640Z-1017 – QIAGEN; Table S2). Targeted sequencing was also performed following one or two cycles of dactinomycin in the BM of 6/10 patients, including 4/4 patients who achieved CR/CRi, and was used to facilitate variant calling at diagnosis.

Ten to 40 ng of input DNA was used for all samples, including one which was preliminarily subjected to whole-genome DNA amplification (GE healthcare) in duplicate reactions. Libraries generated according to the manufacturer’s instructions were sequenced on an Illumina MiSeq instrument for 2x151 cycles, using MiSeq Reagent Kit v3 and V2, to a mean total and unique coverage depth of 11112.42 (range 4811.12-16693.76) and 2045.11 (range 788-5692.33), respectively.

Bioinformatics variant calling was performed with QIAGEN smCounter algorithm with default settings ^9^, and variant annotation was performed with Illumina Variant Studio 3.0. Sequencing variants passing the default filters of smCounter were subjected to the further following filters, and retained only if: i) they were predicted to change the gene coding sequence or involved the conserved splice-site (i.e., the 4 nucleotides surrounding the exon-intron junction); ii) they were present at a variant allele frequency (VAF) of at least 4% in a pre-therapy and/or post-therapy sample; iii) they were not germline polymorphisms (as defined by their presence in both the pre-therapy sample and the matched remission sample at a VAF close to 50% or 100%); iv) they were not present in the Exome Aggregation Consortium (ExAC) database of healthy individuals at a frequency ≥1%. In the only sample previously subjected to whole-genome amplification in duplicate, mutations had also to be present in both replicates. A few variants were observed at an allele frequency >4% in one patient sample but not in the other, and therefore did not satisfy the filter ii described above. However, to allow their tracking across both patient samples, we reported these variants also in samples with <4% variant frequency, as they were supported by the sequencing of more than 25 unique (individually barcoded) mutant molecules originally present in these samples.

**SUPPLEMENTAL REFERENCES**

1 Dohner, H. *et al.* Diagnosis and management of AML in adults: 2017 ELN recommendations from an international expert panel. *Blood* **129**, 424-447, doi:10.1182/blood-2016-08-733196 (2017).

2 Falini, B. *et al.* Cytoplasmic nucleophosmin in acute myelogenous leukemia with a normal karyotype. *The New England journal of medicine* **352**, 254-266, doi:10.1056/NEJMoa041974 (2005).

3 Tiacci, E. *et al.* The NPM1 wild-type OCI-AML2 and the NPM1-mutated OCI-AML3 cell lines carry DNMT3A mutations. *Leukemia* **26**, 554-557, doi:10.1038/leu.2011.238 (2012).

4 Hamaguchi, H. *et al.* Establishment of a novel human myeloid leukaemia cell line (HNT-34) with t(3;3)(q21;q26), t(9;22)(q34;q11) and the expression of EVI1 gene, P210 and P190 BCR/ABL chimaeric transcripts from a patient with AML after MDS with 3q21q26 syndrome. *Br J Haematol* **98**, 399-407, doi:10.1046/j.1365-2141.1997.2143029.x (1997).

5 Chi, H. *et al.* Detection of exon 12 type A mutation of NPM1 gene in IMS-M2 cell line. *Leukemia Research* **34**, 261262, doi:10.1016/j.leukres.2009.09.019 (2010).

6 Quentmeier, H. *et al.* Cell line OCI/AML3 bears exon-12 NPM gene mutation-A and cytoplasmic expression of nucleophosmin. *Leukemia* **19**, 1760-1767, doi:10.1038/sj.leu.2403899 (2005).

7 Brunetti, L. *et al.* Mutant NPM1 Maintains the Leukemic State through HOX Expression. *Cancer Cell* **34**, 499-512 e499, doi:10.1016/j.ccell.2018.08.005 (2018).

8 Martelli, M. P. *et al.* A western blot assay for detecting mutant nucleophosmin (NPM1) proteins in acute myeloid leukaemia. *Leukemia* **22**, 2285-2288, doi:10.1038/leu.2008.149 (2008).

9 Xu, C., Nezami Ranjbar, M. R., Wu, Z., DiCarlo, J. & Wang, Y. Detecting very low allele fraction variants using targeted DNA sequencing and a novel molecular barcode-aware variant caller. *BMC genomics* **18**, 5, doi:10.1186/s12864-016-3425-4 (2017).
