# Supplementary material for: Dactinomycin induces complete remission associated with nucleolar stress response in relapsed/refractory NPM1-mutated AML
Source: Leukemia. 2021 Mar 2;35(9):2552–62. doi: 10.1038/s41375-021-01192-7 (PMC8410589; doi:10.1038/s41375-021-01192-7)

Protocol synopsis

A PHASE II STUDY OF THE CLINICAL ACTIVITY AND SAFETY OF ACTINOMYCIN D IN PATIENTS WITH RELAPSED/REFRACTORY ACUTE MYELOID LEUKEMIA WITH NUCLEOPHOSMIN (NPM1) GENE MUTATION

Trial Code: ActD-AML-PG01
Trial Phase: II
EudraCT number: 2014-000693-18

Study objectives and endpoints

This is a Phase 2 clinical study focused to evaluate the anti-leukemic activity and toxicity of actinomycin D, given as single drug, in adult patients with *NPM1*-mutated AML (age >18 years old) which are refractory to or at relapse after standard intensive chemotherapy and/or hemopoietic stem cell transplantation.

Primary Endpoint

To determine the complete response rate (CR) of *NPM1*-mutated AML patients to the treatment with single drug actinomycin D at the end of the induction arm administration (i.e. after one or two cycles of treatment, according to the time schedule indicated in the study flow chart. Complete response rate is intended as the sum of complete response (CR) and complete response with incomplete marrow recovery (CRi).

Secondary Endpoints

1. To summarize the type, incidence, severity, and relationship to the study-drug of adverse events (AE), severe adverse events (SAE) and any laboratory abnormalities

2. To determine the overall survival (OS) at 1 (one) year, leukemia free survival (LFS) at 1 (one) year, event free survival (EFS) at 1 (one) year and the cumulative incidence of relapse at 1 (one) year

3. To establish the feasibility of allogeneic bone marrow transplantation in patients pretreated with actinomycin D who achieved CR and eligible for allotransplantation

4. To assess time to CTCAE 4.03 grade 4 neutropenia and/or grade 4 thrombocytopenia after either induction or each consolidation cycle
5. To assess time to neutrophil (PMN>1000/μl) and platelet (PLT>50000/μl) recovery after either induction or each consolidation cycle
6. To define the duration of response (assessed from the date of achievement of CR/CRi) in patients not eligible for allotransplantation
7. To investigate the plasma levels of actinomycin D in *NPM1*-mutated AML patients using mass spectrometry analysis
8. To study, in primary *NPM1*-mutated AML blasts, the *in vitro* effect of actinomycin D on nucleoplasmic translocation of the NPM1 protein, on the levels of NPM1 wild-type and mutant proteins, and on survival and apoptosis of leukemic cells

Study design

This is a unicentre, open, phase-2 single arm study of the intravenously administered single drug actinomycin D in relapsed/refractory AML patients harboring *NPM1* mutations.

Actinomycin D will be administered intravenously (in 3-5 minutes) at the dose of 15 µg/Kg/die for 5 consecutive days, at approximately the same time each day.

Administration of actinomycin D for 5 consecutive days followed by an interval of 2-4 weeks (depending on both hematological and extra-hematological toxicity) defined one cycle of therapy. Patients received a total of either five or six cycles of actinomycin D, unless eligible for allotransplant or withdrawn from the study. Specifically, patients in complete remission (CR or CRi) after the first cycle of actinomycin D (induction 1) continued on treatment for additional four cycles (consolidation 1-4) for a total of five cycles. Patients that did not obtain complete remission (CR or CRi) after induction 1 received a second induction cycle (induction 2). Patients in CR after induction 2 received four consolidation cycles with actinomycin D for a total of six cycles. Patients not achieving CR after two induction cycles or demonstrating disease relapse or progression at any time after the 2 (two) induction cycles were withdrawn from the study. Patients who obtain remission (CR/CRi) may be initiated into an allotransplant procedure at any time in the study if they are considered fit and if a bone marrow donor is available.

Patients who obtain remission (CR/CRi) after treatment with Actinomycin D, but relapse after six months from the beginning of the follow-up can be retracted with the same doses, modalities and timing (infusion of 3-5 minutes at the dose of 15 μg/Kg/die) for up to additional 4 (four) cycles.

A pre-treatment with Hydroxyurea is allowed up to 48 hours before the first dose of actinomycin D

Sample Size

Ten (n=10) AML patients harboring *NPM1* mutations, fulfilling the characteristics described in the paragraph "Study population" are planned to be enrolled into this trial during 1.5 years.

Study population

The following categories of *NPM1*-mutated AML patients are eligible for inclusion in this protocol

Principal inclusion criteria

Group 1: Patients (18-60 years-old) with *NPM1*-mutated AML (independently by the mutational status of *FLT3*) whose disease is refractory to or relapses after at least two different lines of therapy

Group 2: Patients (≥60 years old) with *NPM1*-mutated AML (independently by the mutational status of *FLT3*) whose disease is refractory to or relapses after one line of therapy

Other inclusion criteria

Patients must meet all of the following inclusion criteria to be eligible for enrollment into the trial.

1. Proven diagnosis of *NPM1*-mutated AML according to the morphological and immunophenotypic criteria (aberrant cytoplasmic expression of nucleophosmin) of the World Health Organization (WHO-2008) classification of lymphoid neoplasms, accompanied by the presence of *NPM1* mutation as detected using molecular techniques.

2. Patients with refractory/relapsed *NPM1*-mutated AML

3. Any prior treatment (chemotherapy and/or hemopoietic stem cell transplant) must have been completed at least 4 weeks prior to initiation of study medication

4. ECOG PS of 0-2

5. Adequate renal and liver function as defined by the following laboratory values performed within 7 days prior to first dose of actinomycin D: serum creatinine ≤2.0 mg/dl; serum aspartate transaminase (AST) and serum alanine transaminase (ALT) ≤3 times the upper limit of normal (ULN), alkaline phosphatase ≤2.5 times ULN and bilirubin ≤1.5 times the ULN. Higher values are acceptable if they are directly related to the disease

6. Negative serum pregnancy test within 7 days prior to commencement of dosing in premenopausal women. Women of non-childbearing potential may be included if they are either surgically sterile or have been postmenopausal for ≥1 year

7. Fertile men and women must use an effective method of contraception during treatment and for at least 6 months after completion of treatment

8. No psychological, familial, sociological or geographical condition potentially hampering compliance with the study protocol and follow-up schedule

9. Signed informed consent must be obtained prior to performing any study-related procedures

10. Clinical indication for treatment include: morphological and immunohistochemical documentation of disease relapse (≥ 20% bone marrow infiltration by leukemic cells showing aberrant cytoplasmic expression of NPM1); morphologically and immunohistologically documented extramedullary relapse (with the exception of meningeal leukemia involvement, see below) with or without concomitant bone marrow infiltration.

Exclusion Criteria

1. Patients with a previous malignancy within the past 2 years. This criterion does not apply to patients with treated and controlled basal or squamous cell carcinoma of the skin or carcinoma in-situ of the cervix. Isolated elevation in prostate specific antigen (PSA) in absence of radiographic evidence of metastatic prostate cancer is allowed

2. Central nervous system (CNS) leukemia involvement
3. Concurrent administration of any anti-leukemic therapy other than that administered in this study
4. Known hypersensitivity to actinomycin D
5. Pregnant (negative serum pregnancy test is required in women of child-bearing potential) or lactating women
6. Any of the following within the 6 months prior to study drug administration: myocardial infarction, severe/unstable angina, symptomatic congestive heart failure, cerebrovascular accident or transient ischemic attack, pulmonary embolism, hypertension not adequately controlled by current medications
7. Active hepatitis infection or positivity for human immunodeficiency virus
8. Uncontrolled medical illness
9. Other severe, acute or chronic medical or psychiatric condition or laboratory abnormality that may increase the risk associated with study participation or study drug administration, or may interfere with the interpretation of study results,
10. Unwillingness to practice effective birth control
11. Inability to comply with other requirements of the protocol
12. Unwillingness to participate to the study

Definition of response to actinomycin D therapy

For each cycle of therapy, the type of objective response (CR, CRi, PR, NR) will be evaluated between day 15 and 28 after the end of the induction arm administration (i.e. after one or two cycles of treatment, according to the time schedule indicated in the study flow chart shown in Appendix 3). Response to treatment is defined as follows:

The complete response (CR) is defined as the presence at bone marrow examination (bone marrow aspirate and/or bone marrow biopsy) of ≤5% leukemic cells with evidence of trilineage regeneration. In addition, hematological parameters have to show, without growth factor or transfusion support, a normalization of all abnormally low blood counts, i.e. neuthrophils $>1,5x10^9$ per liter, hemoglobin ≥10 g per deciliter and platelets $>100x10^9$

per liter. The definition of CR also applies to complete disappearance of extramedullary masses under the circumstances in which the relapse occurs in an extramedullary site without concomitant bone marrow or peripheral blood involvement.

The complete response with incomplete marrow recovery (CRi) is defined as the presence at bone marrow examination (bone marrow aspirate and/or bone marrow biopsy) of ≤5% leukemic cells with incomplete recovery of hematological parameters as defined by neuthrophils ≤1,5x10^9 per liter, hemoglobin ≤10 g per deciliter and platelets ≤100x10^9 per liter. Physical examination and/or imaging have to demonstrate disappearance of extramedullary masses (if present).

The partial response (PR) is defined as the presence at morphological bone marrow examination (bone marrow aspirate and/or bone marrow biopsy) of a percentage of leukemic cells ranging between 5 and 25% of all bone marrow cells and all hematologic criteria for CR and a decrease of blast percentage of at least 50%. For patients with extramedullary disease, PR definition requires at least a 50% decrease in sum of the product of the diameters of extramedullary masses. Patients who achieve CR or PR in the bone marrow but show partial response of extramedullary disease are regarded as partial responders.

No response (NR) is defined as the presence at bone marrow examination (bone marrow aspirate and/or bone marrow biopsy) of a percentage of leukemic cells ≥20%, independently by the values of blood cell count.

Morphological analysis of both bone marrow biopsy and bone marrow aspirate, as well as bone marrow immunohistochemistry, will be performed by two different specialists. Morphological analysis of bone marrow aspirates will be done counting at least 500 cells.

In patients that, at the time of relapse, show both a medullary and extramedullary involvement by *NPM1*-mutated AML, disappearance, reduction, stability or progression of the extramedullary localizations will be recorded separately from bone marrow response, after each cycle.

Assessment of toxicity to actinomycin D therapy

The patients enrolled in the study will be treated as in-ward patients and monitored daily with complete physical examination and vital signs. The following evaluations will be conducted to assess the safety of the treatment regimen

- Clinical laboratory evaluations (complete and differential blood count; serum concentration of urea nitrogen, creatinine, uric acid, total proteins, albumin, total bilirubin,

AST, ALT, γGT, ALP, LDH, amylase, sodium, potassium, calcium, magnesium, phosphorus, chloride, glucose and fibrinogen; aPTT and PT coagulation analyses or INR value; urine analysis) will be carried out daily

- The electrocardiogram, ecocardiogram and chest X-ray will be evaluated before and at the end of each cycle of therapy, and at any time during the course of treatment, according to clinical need and at the investigator's discretion
- Abdominal ecography or other imaging procedures (CT scan, PET, MNR) will be performed according to clinical need and at the investigator's discretion
- Concomitant medication and procedures
- Adverse events (AEs) related to actinomycin D will be reported according to CTCAE v.4.03 *grading.*

Statistical methods

A Simon's minimax two-stage design will be adopted. This design allows to stop the trial if the minimum required percentage of responses (after one or two induction cycles according to the time schedule indicated in the study flow chart) is not met in a pre-defined fraction of the total number of patients to be enrolled. Otherwise, enrolment continues until the total number of planned patients is reached.

The study parameters are as follows: p_0=0.1, p_1=0.45, α=0.05, β=0.2, n_1=6, n= 10

The null hypothesis (drug not effective) is that the complete remission rate (CR + CRi) at the end of one or two induction cycles is ≤10% (p_0); the alternative hypothesis (drug effective) is that the complete remission (CR - CRi) rate at the end of one or two induction cycles ≥45% (p_1). By enrolling a total of 10 patients (n) and by interim evaluating the remission rate in the first 6 enrolled patients (n_1), the study has 80% probability (1-β) of accepting the alternative hypothesis if the latter is indeed true and 95% probability (1-α) of accepting the null hypothesis if the latter is indeed true.

In particular, according to the two-stage design, if less than 2 complete remissions are observed in the first 6 (n_1) enrolled patients at the end of one or two induction cycles, the drug is declared not effective and accrual is stopped.

Otherwise, the trial continues until enrollment of the total number of planned patients (n=10). If less than 3 complete remissions are then observed at the end of one or two induction cycles, the drug is declared not effective. Conversely, if at least 3 complete remissions are observed in the total of 10 planned patients at the end of one or two induction cycles, the drug is declared effective.

Study duration

The study duration is estimated in about four (4) years and six (6) months including follow-up period and eventual retreatments.

Actinomycin D as single agent in relapsed/refractory AML with mutated *NPM1*
Study protocol flow-chart

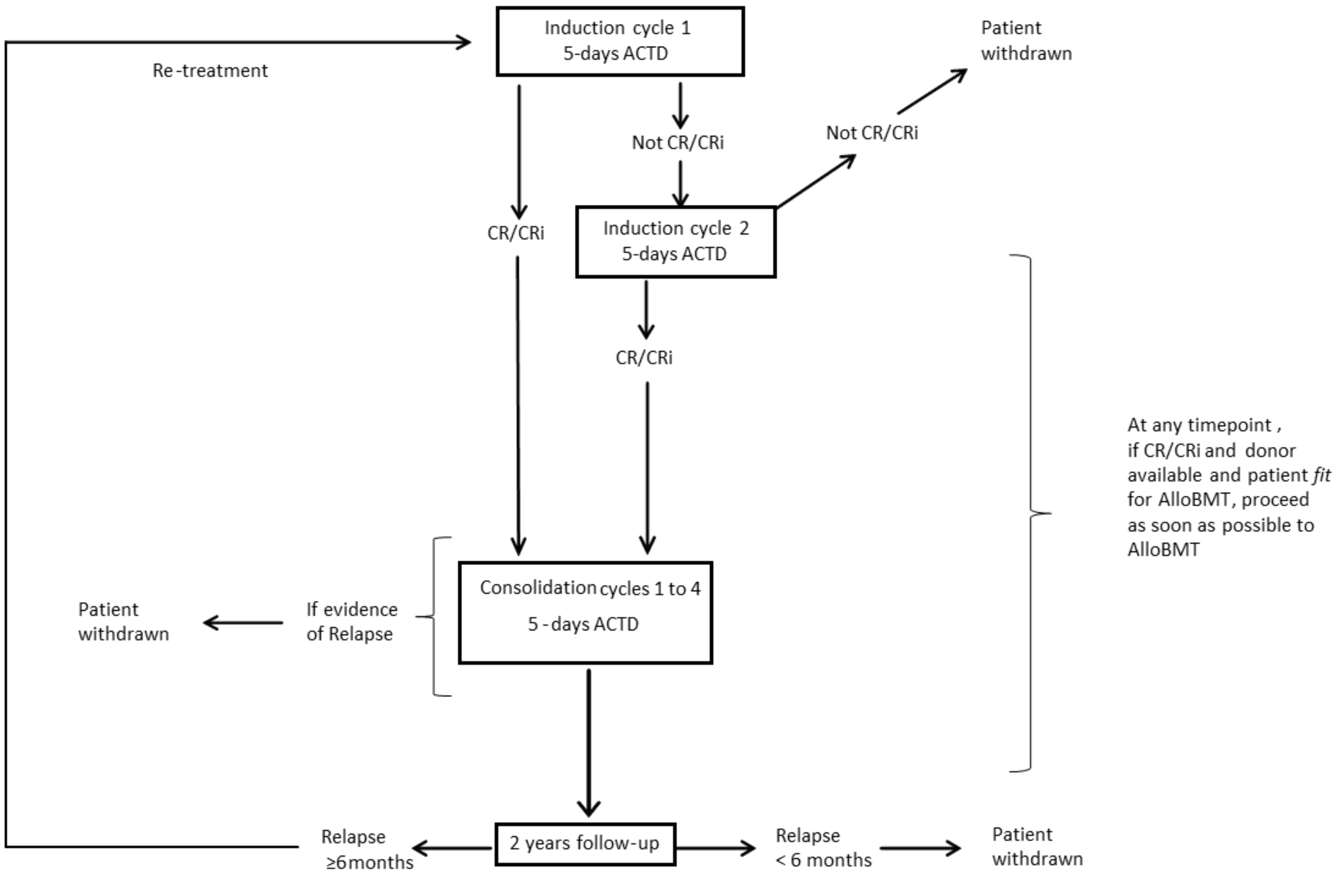

Supplement: Supplementary file 2 — Protocol Synopsis [file 41375_2021_1192_MOESM2_ESM.pdf]
